# Association between RC/HDL-C and hyperuricemia in adults: evidence from NHANES 2005-2018

**DOI:** 10.3389/fendo.2025.1514067

**Published:** 2025-02-24

**Authors:** Yanghao Tai, Bin Chen, Yingming Kong, Xuening Wang

**Affiliations:** ^1^ Third Hospital of Shanxi Medical University, Shanxi Bethune Hospital, Shanxi Academy of Medical Sciences Tongji Shanxi Hospital, Taiyuan, China; ^2^ Department of Cardiovascular Surgery, Shanxi Bethune Hospital, Shanxi Academy of Medical Science, Tongji Shanxi Hospital, Third Hospital of Shanxi Medical University, Taiyuan, China

**Keywords:** hyperuricemia, RC/HDL-C, lipid metabolism, NHANES, population-based studies

## Abstract

**Background:**

The incidence of hyperuricemia is growing in the world, with a significant influence on the survival and healthy condition of the patient. The connection between serum residual cholesterol (RC) to high-density lipoprotein cholesterol (HDL-C) ratio and hyperuricemia is uncertain. Consequently, we tried to elucidate the connection between the hyperuricemia and RC/HDL-C ratio.

**Methods:**

Based on the National Health and Nutrition Examination Survey (NHANES) database, data from 2005 to 2018 were utilized in this cross-sectional research. RC/HDL-C index was calculated by (TC - HDL-C - LDL-C)/HDL-C. Participants were diagnosed with hyperuricemia when the serum uric acid concentration reached 6 mg/dL in women and 7 mg/dL in men. Our researcher utilized smoothed curve fitting and multivariate logistic regression analysis to examine between RC/HDL-C and hyperuricemia among adults. The consistency of these results was examined in various population subgroups.

**Results:**

2376 individuals (19.1%) were stratified into the hyperuricemia group. We observed statistically significant differences (P values < 0.05) in the hyperuricemia population for remaining variables, except for economic level and alcohol drinking. After correcting for potential confounders, our researchers discovered the strong positive connection between the RC/HDL-C and the possibility of incurring hyperuricemia. The incidence of RC/HDL-C elevated by 98% with each additional unit of the RC/HDL-C. Subgroup analyses showed correlations for the majority of subgroups remained stable. However, gender and several diseases may modify this association.

**Conclusions:**

Higher RC/HDL-C is correlated with higher prevalence rate of developing hyperuricemia. However, further research is still required to confirm the causal association.

## 1 Introduction

Hyperuricemia is a chronic metabolic disease caused by excessive uric acid production or insufficient excretion, which leads to an abnormal increase in the uric acid level in serum and is characterized by purine metabolism disorder (1). It is a worldwide issue related to public health, connected with a wide range of other conditions, affecting people of different ages, and has been trending towards a younger age group in recent years (2, 3). Several recent findings have revealed the prevalence of hyperuricemia appears to be on the rise worldwide. The incidence of hyperuricemia is about 20% in the United States (4). Hyperuricemia is often preceded by gouty attacks and gout stone formation, which greatly reduces people’s quality of life, although asymptomatic patients do exist (5). However, even in asymptomatic patients, hyperuricemia remains an independent risk factor for metabolic syndrome, hypertension, many chronic kidney diseases, and even death (6–9), which seriously jeopardizes people’s health. Uric acid represents the terminal metabolite of purine catabolism in humans, with approximately two-thirds derived from endogenous purine metabolism and the remaining one-third attributable to the ingestion of purine-rich dietary sources (10, 11). The absence of urate oxidase in humans precludes the further metabolic breakdown of uric acid into the more water-soluble allantoin, thereby elevating serum uric acid levels approximately five- to sixfold higher than those observed in other mammals (12). The renal system is the predominant route for uric acid excretion, accounting for approximately 70% of total clearance, while the remaining 30% is cleared through extra-renal mechanisms (13, 14). Within the kidneys, specific uric acid transporters, such as URAT1 (urate transporter 1) and GLUT9 (glucose transporter 9), are instrumental in mediating the reabsorption and secretion of uric acid, thereby regulating its overall renal handling (15).

Low-density lipoprotein cholesterol (LDL-C) is undoubtedly a critical component in the lipoprotein profile. However, with the popularity of statins, the focus of research has shifted to triglyceride-rich lipoproteins (TRL), which are the hallmark component of dyslipidemia and are not affected by statins (16, 17). Residual cholesterol (RC), which reflects the level of cholesterol in triglyceride-rich lipoproteins (TRLs), is an emerging nontraditional marker of lipids and consists of celiac remnants, intermediate-density lipoproteins (IDLs), and very-low-density lipoproteins (VLDLs) (18). Actually, RC is essentially a form of cholesterol, and studies have shown that elevated levels of cholesterol in triglyceride-rich lipoproteins (TRLs) pose a more direct and significant risk to the incidence of cardiovascular and metabolic diseases than traditional lipid markers such as LDL-C & TG (19–21). Although TG levels are often used as a clinical surrogate for TRLs or RC, increasing emphasis is being placed on RC in current lipid management practices (22). The metabolism of RC primarily involves the synthesis, transport, and catabolism of lipids, with its levels being influenced by multiple factors, including dietary fat intake, hepatic lipid synthesis, and lipoprotein metabolism (23). Elevated RC levels have been associated with increased cardiovascular risk, as these lipoproteins can penetrate the endothelium and contribute to atherosclerotic plaque formation (24).

There is a marked divergence in the metabolic pathways of residual cholesterol and uric acid. The metabolism of uric acid is predominantly centered around the purine metabolic pathway, with its production and excretion regulated by enzymes and transport proteins in the liver and kidneys (25). In contrast, the metabolism of residual cholesterol encompasses the synthesis, transport, and breakdown of lipoproteins, which are primarily governed by lipid metabolic pathways in the liver and small intestine (26). Despite their distinct metabolic pathways, both are closely associated with metabolic syndrome and cardiovascular diseases (27). The ratio of Residual Cholesterol to High-Density Lipoprotein Cholesterol (RC/HDL-C) is an important indicator of lipid metabolite interactions, reflecting the balance between atherogenic and anti-atherogenic lipoproteins (28). Thus, exploring the potential connections between the two holds significant clinical importance. In recent years, with the increasing research on proteomics, metabolomics and genomics, many molecular markers are emerging to provide important information for disease diagnosis, prognosis and therapeutic targets (29). However, limited research has been conducted on the correlation between increased RC/HDL-C and the likelihood of hyperuricemia, particularly in the broader US population. We conducted the following cross-sectional study using data related to the 2005-2018 National Health and Nutrition Examination Survey (NHANES) to prove the connection with RC/HDL-C and hyperuricemia.

## 2 Materials and methods

### 2.1 Data sources

The 2005-2018 NHANES datasets were the original information resource for the present analysis. The NHANES database provides a comprehensive range on each participant’s laboratory tests, disease information, human examinations, and population characteristics. The reliability of the data in this database stems from the fact that it is collected based on the nationwide representative sample in United States via a comprehensive investigation executed, the use of the most advanced and reliable techniques of information collection and organizational and statistical methods for data analysis. This data is subsequently subjected to additional analysis and utilized to study risk factors for a variety of diseases. A sophisticated multi-stage probabilistic approach was used in this study to ensure the representativeness and accuracy of the samples used. The qualification of human subjects in NHANES was approachable via the National Center for Health Statistics (NCHS) Ethics Review Board and each individual signed up an informed agreement.

### 2.2 Research population

**Figure 1:**
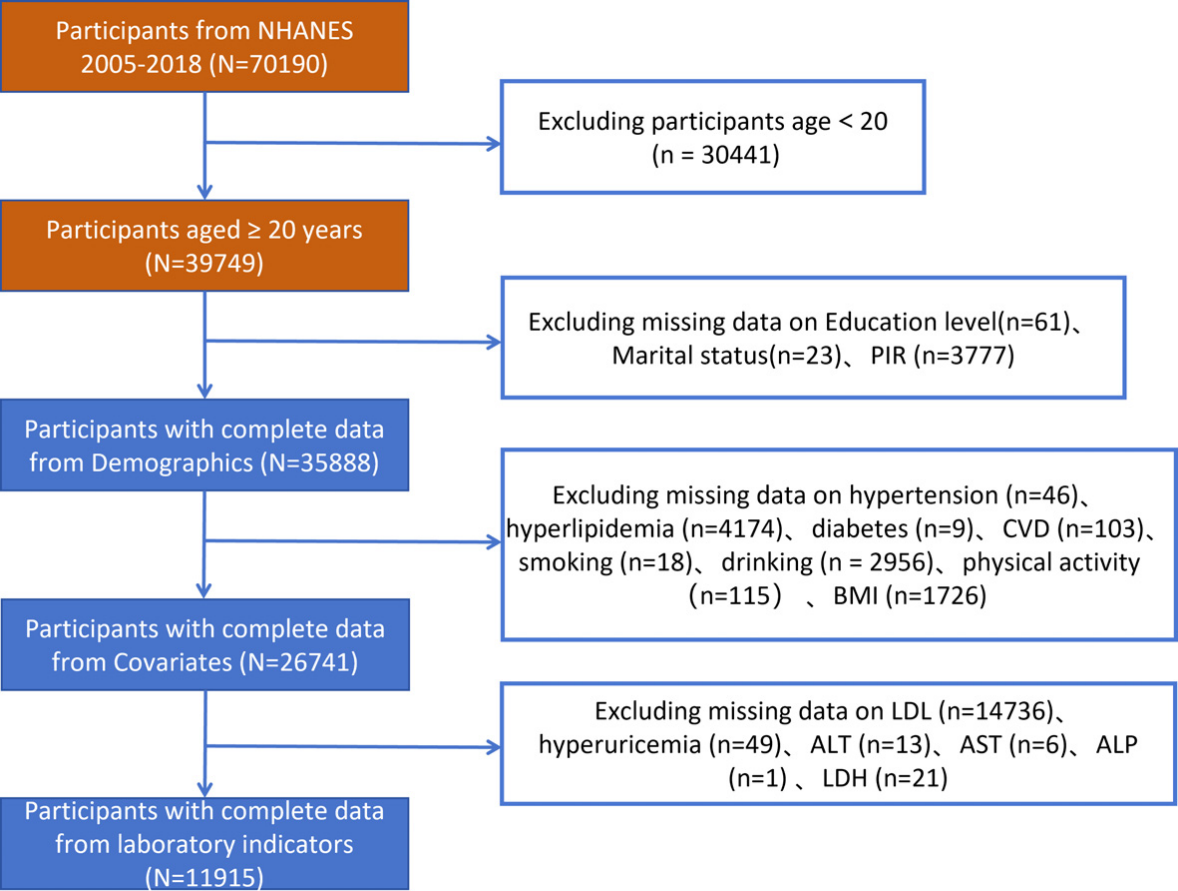
Flowchart depicting participant selection in the study.

The researchers selected 70190 participants from NHANES 2005-2018.The researchers requested that the dataset be a usable dataset containing information on hyperuricemia and relevant variables needed to calculate the RC/HDL-C. The researchers applied the exclusion criterion to the following groups to: participants aged <20 years, lack of socio-demographic data, confounders, and incomplete information on hyperuricemia, and participants with incomplete data required for RC/HDL-C calculations. A total of 11915 participants with comprehensive information participated in this cross-sectional investigation after exclusion of participants who met the above criteria (**Figure 1**).

### 2.3 Definition of exposed variables

RC/HDL-C index (30) was regarded in the current investigation as an exposure variable, calculated by (TC - HDL-C - LDL-C)/HDL-C. Their unit is the mmol/L. On a Cobas 6000 biochemistry instrument, high-density lipoprotein cholesterol (HDL-C) values were assessed. We treated the RC/HDL-C index as the categorical variable and the continuous one for correlation studies with the intention of further investigate the connection between hyperuricemia and the RC/HDL-C.

### 2.4 Definition of the outcome variable

The researchers considered the prevalence of hyperuricemia as an outcome variable. According to the current studies (31), the diagnosis of hyperuricemia was diagnosed by uric acid levels. Participants were defined with hyperuricemia when serum uric acid concentration reached 7 mg/dL in men and 6 mg/dL in women. Experts examined the levels of serum uric acid using a Roche Cobas 6000 analyzer.

### 2.5 Covariates

Factors that may have an impact on the connection between hyperuricemia and the RC/HDL-C were regarded as covariates in this investigation. Covariates considered in this study included socio-demographic factors including age (≤40, 41-60, >60), educational level (high school graduate/GED or equivalent, college graduate or above, less than 12th grade), ethnicity (other Hispanic, other races, Mexican American), family poverty income ratio (PIR) (≤1.0, 1.1-4.0, >4.0), and marital condition (living alone, married and living with partner). The researchers also included health related factors, diabetes, physical activity, hypertension, hyperlipidemia, cardiovascular diseases (all described as yes/no). PIR is the measurement of socioeconomic position, defined as the ratio of income to the United States Census Bureau’s poverty level for a family. Individuals are classified as present smokers, never smokers, and former smokers based on “Do you personally use cigarette or tobacco?” and “Smoked more than 100 cigarettes in your lifetime”. Based on “Drink at least twelve times in a lifetime or a year”, the definition of a drinker includes current, never, and former drinkers. According to questionnaire information, self-reported disease was also considered, including diabetes. Cardiovascular disease, hypertension, and hyperlipidemia were diagnosed on the basis of self-reported history of specific diseases. The description of cardiovascular disease includes heart attack, stroke, congestive heart failure, angina, and coronary heart disease. The specialized medical technicians take careful measurements of an individual’s weighted and standing height in Mobile Examination Center. Laboratory data include liver enzymes, lipids, lactate dehydrogenase, and creatinine.

### 2.6 Statistical methods

Continuous and categorical variables were used to characterize participants at baseline. Continuous ones are described in terms of mean ± standard deviation and categorical variables are described by numbers and percentages. Weighted t-tests and chi-square tests were conducted for comparisons of baseline characteristics among these participants. By assessing the corrected OR and 95% CI, the study conducted the weighted logistic regression model, to investigate the connection between RC/HDL-C and hyperuricemia. Three models were used by us with varying degrees of modification for covariates (Model 1, unadjusted for covariates; Model 2, modified for gender, education, ethnicity, marital status, PIR, and age; and Model 3, with adjustments for gender, marital status, education, PIR, physical activity, age, alcohol consumption, ethnicity, smoking status, hypertension, cardiovascular disease, hyperlipidemia, diabetes, and laboratory test indicators). In all three models, continuous variable RC/HDL-C was used by our researchers. In addition, the dose-response association between the RC/HDL-C index and the prevalence of the hyperuricemia was evaluated by utilizing the restricted cubic spline (RCS). The multiple logistic regression models included the continuous and categorical models. The RC/HDL-C was divided into quartiles, and then the linear trends were conducted via considering the median value of every subgroup as the continuous variable. In addition, our researchers conducted the subgroup analyses by general information, disease condition, alcohol consumption, smoking status, physical activity, and interaction analyses were performed to examine whether there were different associations between subgroups. All statistical analyses in this investigation were conducting by utilizing R 4.3.3 and SPSS 26.0. The bilateral P value of less than 0.05 was recognized to be statistically significant.

## 3 Result

### 3.1 Baseline characteristics of participants

The study recruited 70190 individuals from NHANES 2005-2018. The flowchart for the exclusion and inclusion of individuals is revealed in **Figure 1**. Participants with incomplete information on age <20 years, socio-demographic data, and covariates were excluded, and the remaining 26741 participants were retained. In addition, after excluding participants who had incomplete data required for RC/HDL-C calculations and hyperuricemia, the remaining 11915 participants were considered in our investigation. The characteristics of participants involved in this investigation at baseline were presented in **Table 1**. 2376 individuals (19.1%) were stratified into the hyperuricemia group. We observed statistically significant differences (P values < 0.05) in the hyperuricemia population for remaining variables, except for economic level and alcohol drinking.

**Table 1:** Baseline characteristics of participants based on hyperuricemia.

Characteristics	N	Participant No. (weighted, %)		P Value
		With hyperuricemia	Without hyperuricemia	
N	11915	2376 (19.1%)	9539 (80.9%)	
Gender				<0.001
Male	5767	1253 (53.4%)	4514 (46.9%)	
Female	6148	1123 (46.6%)	5025 (53.1%)	
Age				<0.001
20-40	3677	541 (27.4%)	3136 (34.7%)	
41-60	4147	766 (36.5%)	3381 (40.6%)	
61-80	4091	1069 (36.1%)	3022 (24.7%)	
Race				<0.001
Mexican American	1576	217 (4.8%)	1359 (7.6%)	
Other Hispanic	1114	173 (3.9%)	941 (5.3%)	
Other Race	9225	1986 (91.3%)	7239 (81.7%)	
Education level				0.011
Less than 12th grade	2522	476 (12.7%)	2046 (13.9%)	
High school graduate/GED or equivalent	2669	585 (24.1%)	2084 (22.1%)	
College graduate or above	6724	1315 (63.1%)	5409 (64.0%)	
Marital status				0.028
Married and living with partner	7346	1418 (64.4%)	5928 (66.3%)	
Living alone	4569	958 (35.6%)	3611 (33.7%)	
PIR				0.428
≤1.0	2269	432 (12.0%)	1837 (12.8%)	
1.1-4.0	6338	1287 (49.5%)	5051 (48.7%)	
> 4.0	3308	657 (38.5%)	2651 (38.5%)	
Smoking				< 0.001
Never	6553	1227 (51.1%)	5326 (55.5%)	
Former	3136	755 (33.0%)	2381 (25.5%)	
Current	2226	394 (15.9%)	1832 (19.0%)	
Drinking				0.074
Never	1572	305 (9.8%)	1267 (10.1%)	
Former	1851	405 (14.4%)	1446 (12.5%)	
Current	8492	1666 (75.8%)	6826 (77.4%)	
Diabetes				<0.001
Yes	1644	460 (14.9%)	1184 (9.0%)	
No	9968	1833 (81.6%)	8135 (89.0%)	
Pre-diabetes	303	83 (3.5%)	220 (2.1%)	
Hypertension				<0.001
Yes	4706	1423 (54.7%)	3283 (30.6%)	
No	7209	953 (45.3%)	6256 (69.4%)	
Hypercholesterolemia				<0.001
Yes	4632	1094 (44.6%)	3438 (35.0%)	
No	7383	1282 (55.4%)	6101 (65.0%)	
CVD				<0.001
Yes	1431	451 (15.0%)	980 (8.4%)	
No	10484	1925 (85.0%)	8559 (91.6%)	
Vigorous recreational activities				< 0.001
Yes	2719	404 (19.6%)	2315 (28.5%)	
No	9196	1972 (80.4%)	7224 (71.5%)	
Moderate recreational activities				< 0.001
Yes	5189	950 (46.3%)	4239 (49.1%)	
No	6726	1426 (53.7%)	5300 (50.9%)	
BMI	11915	32.78 ± 8.02 (32.86 ± 7.78)	28.51 ± 6.48 (28.39 ± 6.44)	<0.001
Albumin	11915	41.73 ± 3.47(42.10 ± 3.38)	41.90 ± 3.49 (42.22 ± 3.40)	0.031
ALT	11915	27.96 ± 20.52 (29.33 ± 20.33)	23.80 ± 16.24 (23.88 ± 15.34)	<0.001
AST	11915	27.27 ± 16.45 (27.41 ± 15.19)	24.60 ± 19.45 (24.31 ± 16.40)	<0.001
ALP	11915	72.26 ± 24.28 (70.75 ± 24.50)	69.73 ± 25.43 (67.53 ± 24.29)	<0.001
BUN	11915	5.94 ± 3.04 (5.71 ± 2.64)	4.68 ± 1.80 (4.70 ± 1.67)	<0.001
CR	11915	93.01 ± 49.09 (89.30 ± 44.61)	76.80 ± 40.18 (75.88 ± 31.44)	<0.001
RC/HDL-C	11915	0.64 ± 0.45 (0.66 ± 0.44)	0.47 ± 0.38 (0.46 ± 0.38)	<0.001

Continuous variables were presented as mean with standard deviation (mean t S), and categorical variables were expressed as proportion. Continuous variables were analyzed via one-way ANOVA; categorical variables were analyzed using the Chi-square test or the Fisher’s exact test, and P-value less than 0.05 was considered statistically significant.

### 3.2 Association between RC/HDL-C and hyperuricemia

This crude mode showed that the prevalence of developing hyperuricemia raised by 1.55 times (OR 2.55, 95% CI 2.30-2.83, P < 0.001) for each unit increase in RC/HDL-C. Model 2 adjusted for socio-demographic factors (marital status, poverty rate, race, age, and education level) and showed a 1.71 (OR 2.71, 95%CI 2.43-3.02, P < 0.001) addition in occurrence of hyperuricemia. Model 3 further controlled for disease status, with every unit addition of the RC/HDL-C significantly increasing the prevalence of hyperuricemia by 98.0% (OR 1.98 95% CI 1.76-2.24, P < 0.001).

**Figure 2:**
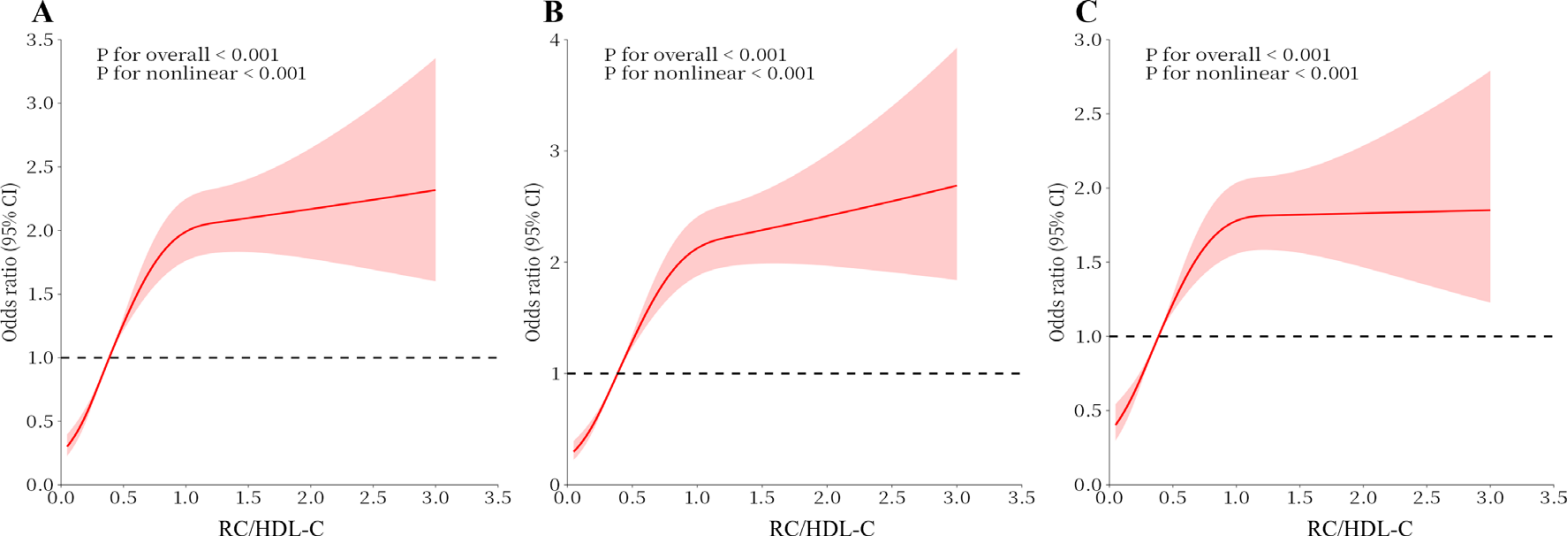
Analysis of the Measured Response Relationship Between RC/HDL-C and hyperuricemia. **(A)** Model 1 was adjusted for none. **(B)** Model 2 was adjusted for age, gender, race, education level, marital status and PIR. **(C)** Model 3 was adjusted for age, race, gender, education level, marital status, PIR, physical activity, smoking status, alcohol use, disease, and Laboratory test indicators. The solid red line indicates the OR and the red shaded area indicates the 95% CI.

As shown in **Table 2**, every added unit of the RC/HDL-C was also categorized by quartile and compared to the first quartile as the reference. In the crude model, compared with the lowest Q1, the highest Q4 of the RC/HDL-C had a 2.82-fold prevalence of hyperuricemia (OR 3.82, 95% CI 3.31-4.40). In Model 2, after an adaptation for socio-demographic considerations, the risk of disease was raised by a factor of 3.10-fold (OR 4.10, 95% CI 3.54-4.74) compared with Q1. In Model 3, the incidence was increased 1.83-fold, after the totally adjustment (OR 2.83, 95% CI 2.40-3.32). In all three models, a remarkable dose-response trend was presented with the addition in the RC/HDL-C index (P for trend < 0.01). The RCS curve constructed the measured response association between RC/HDL-C and hyperuricemia in **Figure 2**.

**Table 2:** Weighted regression models and trend tests elucidating the association between RC/HDL-C and prevalence of hyperuricemia.

RC/HDL-C	Hyperuricemia OR (95% CI)					
	Model 1	P Value	Model 2	P Value	Model 3	P Value
Continuous	2.55 (2.30-2.83)	<0.001	2.71 (2.43-3.02)	<0.001	1.98 (1.76-2.24)	<0.001
Quantile						
Q1	1.00 (Reference)		1.00 (Reference)		1.00 (Reference)	
Q2	1.66 (1.43-1.94)	<0.001	1.67 (1.43-1.95)	<0.001	1.47 (1.25-1.73)	<0.001
Q3	2.62 (2.26-3.03)	<0.001	2.68 (2.31-3.11)	<0.001	2.10 (1.79-2.47)	<0.001
Q4	3.82 (3.31-4.40)	<0.001	4.10 (3.54-4.74)	<0.001	2.83 (2.40-3.32)	<0.001
P for trend		<0.001		<0.001		<0.001

Model 1 was adjusted for none.

Model 2 was adjusted for gender, age, race, education level, marital status and PIR.

Model 3 was adjusted for gender, age, race, education level, marital status, PIR, BMI, physical activity, smoking status, alcohol use, disease, and Laboratory test indicators.

### 3.3 Subgroup analysis

Analyzed by weighted logistic regression, the relationship between hyperuricemia and RC/HDL-C exposure for each stratified indicator are reported in **Table 3**, **Supplementary Table 1**. For each unit addition in RC/HDL-C, the prevalence of hyperuricemia aggrandized by 1.78-fold in female participants (OR 2.78, 95% CI 2.24-3.45). However, for male participants, the prevalence of hyperuricemia increased by 65%, with the increase of the per unit of RC/HDL-C. In terms of education levels, participants those graduated from college or above had a 2.22-fold risk of incidence of hyperuricemia (OR 2.22, 95% CI 1.88 – 2.62). Other high school graduate/GED or equivalent (OR 1.91, 95% CI 1.49 - 2.45) and less than 12th grade (OR 1.63, 95% CI 1.29 – 2.06) also had higher odds. The results of the stratified analysis were reliably stable between remaining socio-demographic groups. The highest risks were observed among participants aged 61-80 years (OR 2.01, 95% CI 1.64 – 2.47), those of other races (OR 2.13,95% CI 1.86 – 2.43), individuals living alone (OR 2.04, 95% CI 1.67 – 2.50), and those with a PIR ≤1.0 (OR 2.14, 95% CI 1.65 – 2.77). Regarding smoking and alcohol consumption, the risk was 2.10-fold for participants who had currently smoked (OR 2.09, 95% CI 1.64 - 2.66) and 2.05-fold for participants who currently consumed alcohol (OR 2.05, 95% CI 1.79 - 2.36). However, we observed remarkable exchanges in the diabetes and hypertension. The tiered analysis of disease status showed 2.22-fold higher addition for participants without hypertension (OR 2.22, 95% CI 1.86-2.66) and 76% elevated risk for individuals with hypertension (OR 1.76, 95% CI 1.50 - 2.06). A favorable association between the RC/HDL-C and hyperuricemia was demonstrated in participants with pre-diabetes (OR 3.03, 95% CI 1.53 - 5.98). However, the statistical relationship between RC/HDL-C and hyperuricemia remained stable among participants who self-reported CVD and hyperlipoidemia.

**Table 3:** Subgroup analysis of the association between RC/HDL-C and hyperuricemia.

Subgroup	[OR (95% CI)]	P Value	P for Interaction
Gender			<0.001
Male	1.65 (1.43 ~ 1.91)	<0.001	
Female	2.78 (2.24 ~ 3.45)	<0.001	
Education level			0.003
Less than 12th grade	1.63 (1.29 ~ 2.06)	<0.001	
High school graduate/GED or equivalent	1.91 (1.49 ~ 2.45)	<0.001	
College graduate or above	2.22 (1.88 ~ 2.62)	<0.001	
Hypertension			<0.001
Yes	1.76 (1.50 ~ 2.06)	<0.001	
No	2.22 (1.86 ~ 2.66)	<0.001	
Diabetes			<0.001
Yes	1.47 (1.11 ~ 1.94)	0.007	
No	2.07 (1.81 ~ 2.38)	<0.001	
Pre-diabetes	3.03 (1.53 ~ 5.98)	0.001	

## 4 Discussion

Our investigation primarily examined the association between the prevalence of hyperuricemia and RC/HDL-C index and using the NHANES database. The research involved in a sample size of 11915 individuals. Weighted multifactorial logistic regression analysis revealed, after all covariates with control, the positive and statistically significant association observed between RC/HDL-C and hyperuricemia. Besides, RCS curves revealed a non-linear positive correlation between hyperuricemia and RC/HDL-C. In addition, the incidence of hyperuricemia progressively elevated with rising quartiles of RC/HDL-C. The statistical association between RC/HDL-C and hyperuricemia remained stable among participants who self-reported CVD and hyperlipidemia. To be emphasized, subgroup analyses revealed that the relevance between RC/HDL-C and hyperuricemia might be altered because of gender, educational levels, hypertension and diabetes.

In the adult population, a growing body of research suggests that hyperuricemia is a frequent condition. Hyperuricemia has a significant influence on the quality of survival and healthy condition of the patient, as well as a remarkable influence on the psychological well-being of individuals. The positive correlation of RC/HDL on the occurrence of hyperuricemia varies in certain subgroups, firstly, at the gender level, there are differences in lifestyle between males and females, where males have a tendency to be regular smokers and drinkers of alcohol, habits that have a negative impact on lipid levels. In addition to this, estrogen may increase HDL-C levels, while androgens may decrease HDL-C levels (32). Secondly, differences in education levels may affect an individual’s lipid levels, and thus the prevalence of hyperuricemia, through a number of pathways. In general, well-educated individuals may have a better understanding of health, including awareness of hyperuricemia and dyslipidemia. They may be more aware of how to manage these health problems through diet, exercise, and other lifestyle changes (33). Education level is often correlated with economic income, which may affect an individual’s ability to access healthy foods and healthcare resources. Individuals with higher educational attainment typically possess greater financial capacity to procure nutritious foods and secure routine health checkups. Moreover, hypertension and diabetes are commonly associated with metabolic syndrome, which can precipitate dyslipidemia, including alterations in HDL-C levels. Dyslipidemia itself can induce vascular wall damage, subsequently leading to elevated blood pressure (34, 35). Collectively, these conditions can contribute to an unstable prevalence RC/HDL-C) relative to hyperuricemia.

However, the pathophysiological mechanisms of hyperuricemia have not yet been fully elaborated. Although the prevalence of hyperuricemia in the U.S. population has remained stable over the last decade, the overall incidence and public health impact of the condition remains quite significant given the rising population size (36), which may indirectly reflect trends in the development of hyperuricemia in other countries around the globe. This exacerbates the strain on healthcare systems and increases the direct and indirect healthcare costs associated with the condition. In addition, hyperuricemia is connected to varieties of chronic diseases including cardiovascular and kidney disease, further increasing the urgency for public health interventions. Elevated RC/HDL-C, a crucial indicator of lipid metabolite interactions, has been associated with the prevalence of hyperuricemia, and this relationship may involve multiple mechanisms. First, studies have shown that dyslipidemia may lead to elevated blood uric acid levels, which increase with increasing TG levels and decreasing HDL levels (37). In the obese state, purine catabolism is enhanced in the body due to increased adipose tissue, leading to increased uric acid production, as has been demonstrated in mouse model studies (38). And the Western dietary pattern is characterized by its composition of high fats and carbohydrates, which may lead to an increased inflammatory response in the body and elevation of various lipid metabolites (39, 40). Based on the analysis of large-scale population statistics, dietary inflammation holds a pivotal position for serum uric acid levels and significantly influences the progression of health status in hyperuricemia (41). Secondly, oxidative stress in adipose tissue is the principally etiological factor for metabolic syndrome and obesity-related inflammation (42, 43). In the pathogenesis of metabolic disorders, for instance, hypertriglyceridemia and nonalcoholic fatty liver disease, hepatic fatty acid oxidation may induce hypoxia-inducible factor-1α (HIF-1α), which in turn transcriptionally activates xanthine dehydrogenase (XDH) and cytoplasmic-5´-nucleotidase-II (NT5C2) in the uric acid synthesis pathway and promotes hepatic uric acid synthesis (43, 44). In addition, the kidneys perform a crucial function in sustaining the homeostasis of uric acid in the body and are responsible for the majority of uric acid excretion, with approximately 70% of uric acid excreted in urine throughout the kidneys (45). Abnormal lipid profile can lead to renal pathology, cholesterol can be ectopically deposited in the kidney, and the presence of cholesterol accumulation in the podocytes without effective intervention will induce oxidative stress, skeletal disorders, and mitochondrial function abnormalities in the podocytes, which will directly activate autophagy of the podocytes, cause the apoptosis of podocyte, and accelerate the damage of glomerular filtration barrier (46). Moreover, dyslipidemia increases the disease susceptibility of coronary heart disease and accelerates the onset of atherosclerosis, as well as being a potential hazard to the kidneys. Elevated lipids can damage the kidneys, leading to enlarged glomeruli, changes in renal tissue, and even glomerulosclerosis, which ultimately affects the excretion of uric acid and accelerates the progression of hyperuricemia under the combined effect of several mechanisms (47). Currently, substantial research has confirmed that when cholesterol levels in triglyceride-rich lipoproteins (TRLs) are elevated, the effect on the development of cardiovascular and other diseases has a more pronounced and direct correlation (48).

According to studies in recent years, the current rate of obesity in the global population continues to rise, affecting people’s physical and mental health in various ways. The universal prevalence of hyperuricemia also keeps escalating year by year, and lipid metabolism is closely associated with hyperuricemia problems. Our investigation confirms a novel demonstration of a remarkable association between the occurrence of hyperuricemia and RC/HDL-C, which has significant implications for the future diagnosis as well as treatment of hyperuricemia. The present study has some strengths, based on the NHANES database, the investigation is the primarily cross-sectional investigation to research the correlation between RC/HDL-C and hyperuricemia. At the same time, our study is representative because it contains basic data from a large number of US respondents. However, it is worth noting that we still have some limitations, firstly some of the disease information in the NHANES database was obtained through self-reporting by the respondents, including disease information. And the self-reporting deviation could lead to imprecise messages, which may affect the accuracy of the results. Second, this investigation could not establish the direct sequential association between hyperuricemia and RC/HDL-C, but only inferred a correlation, due to the cross-sectional study. In conclusion, this study suggests that there is the significantly positive relationship between the hazard of hyperuricemia and the RC/HDL-C.

## 5 Conclusions

The research revealed the positively remarkable relationship between hyperuricemia and RC/HDL-C in the American population. Higher RC/HDL-C is associated with higher prevalence of hyperuricemia. The founding suggests that lipid metabolism might be an influenced factor for hyperuricemia. The clinical importance of our research is that assessing RC/HDL-C may help identify those at higher prevalence for hyperuricemia. Incorporation of the RC/HDL-C into common clinical assessment could contribute to inchoate detection of hyperuricemia and direct personalized care strategies.

## Data Availability

Publicly available datasets were analyzed in this study. This data can be found here: NHANES data used in this work are publicly available. All raw data are available on the NHANES website (https://www.cdc.gov/nchs/nhanes/). Further inquiries can be directed to the corresponding author.
