# Supplementary material for: Association between RC/HDL-C and hyperuricemia in adults: evidence from NHANES 2005-2018
Source: Front Endocrinol (Lausanne). 2025 Feb 24;16:1514067. doi: 10.3389/fendo.2025.1514067 (PMC11891044; doi:10.3389/fendo.2025.1514067)
Supplement: Supplementary file 1 [file Table1.docx]

Supplementary Table 1. Subgroup analysis of the association between RC/HDL-C and hyperuricemia.

| Subgroup | [OR (95% CI)] | P Value | P for Interaction |
| --- | --- | --- | --- |
| **Age** |  |  | 0.401 |
| 20-40 | 1.87 (1.48 ~ 2.37) | <0.001 |  |
| 41-60 | 1.98 (1.63 ~ 2.42) | <0.001 |  |
| 61-80 | 2.01 (1.64 ~ 2.47) | <0.001 |  |
| **Race** |  |  | 0.053 |
| Mexican American | 1.43 (1.04 ~ 1.98) | 0.029 |  |
| Other Hispanic | 1.87 (1.21 ~ 2.89) | 0.005 |  |
| Other Race - Including Multi-Racial | 2.13 (1.86 ~ 2.43) | <0.001 |  |
| **Marital status** |  |  | 0.878 |
| Married and living with partner | 1.95 (1.68 ~ 2.26) | <0.001 |  |
| Living alone | 2.04 (1.67 ~ 2.50) | <0.001 |  |
| **PIR** |  |  | 0.933 |
| ≤1.0 | 2.14 (1.65 ~ 2.77) | <0.001 |  |
| 1.1-4.0 | 2.04 (1.73 ~ 2.40) | <0.001 |  |
| >4.0 | 1.87 (1.47 ~ 2.39) | <0.001 |  |
| **CVD** |  |  | 0.422 |
| No | 1.97 (1.73 ~ 2.25) | <0.001 |  |
| Yes | 1.96 (1.46 ~ 2.62) | <0.001 |  |
| **Vigorous recreational activities** |  |  | 0.502 |
| Yes | 1.82 (1.36 ~ 2.43) | <0.001 |  |
| No | 2.01 (1.76 ~ 2.29) | <0.001 |  |
| **Moderate recreational activities** |  |  | 0.330 |
| Yes | 1.96 (1.60 ~ 2.39) | <0.001 |  |
| No | 2.02 (1.73 ~ 2.34) | <0.001 |  |
| **Hypercholesterolemia** |  |  | 0.118 |
| Yes | 1.97 (1.65 ~ 2.35) | <0.001 |  |
| No | 1.97 (1.67 ~ 2.32) | <0.001 |  |
| **Smoking** |  |  | 0.221 |
| Never | 2.15 (1.79 ~ 2.57) | <0.001 |  |
| Former | 1.83 (1.48 ~ 2.26) | <0.001 |  |
| Current | 2.09 (1.64 ~ 2.66) | <0.001 |  |
| **Drinking** |  |  | 0.225 |
| Never | 1.91 (1.29 ~ 2.82) | 0.001 |  |
| Former | 1.75 (1.25 ~ 2.45) | 0.001 |  |
| Current | 2.05 (1.79 ~ 2.36) | <0.001 |  |
